# Effect of Laser Scan Speed on Defects and Texture Development of Pure Chromium Metal Fabricated via Powder Bed Fusion-Laser Beam

**DOI:** 10.3390/ma17092097

**Published:** 2024-04-29

**Authors:** Yong Seong Kim, Ozkan Gokcekaya, Aira Matsugaki, Takayoshi Nakano

**Affiliations:** 1 Division of Materials and Manufacturing Science, Graduate School of Engineering, Osaka University, 2-1 Yamadaoka, Suita 565-0871, Osaka, Japan; yongseong.kim@mat.eng.osaka-u.ac.jp (Y.S.K.); matsugaki@mat.eng.osaka-u.ac.jp (A.M.); 2 Anisotropic Design & Additive Manufacturing Research Center, Osaka University, 2-1 Yamadaoka, Suita 565-0871, Osaka, Japan

**Keywords:** chromium, powder bed fusion-laser beam, densification, texture, cracking

## Abstract

Chromium (Cr) metal has garnered significant attention in alloy systems owing to its exceptional properties, such as a high melting point, low density, and superior oxidation and corrosion resistance. However, its processing capabilities are hindered by its high ductile–brittle transition temperature (DBTT). Recently, powder bed fusion-laser beam for metals (PBF-LB/M) has emerged as a promising technique, offering the fabrication of net shapes and precise control over crystallographic texture. Nevertheless, research investigating the mechanism underlying crystallographic texture development in pure Cr via PBF-LB/M still needs to be conducted. This study explored the impact of scan speed on relative density and crystallographic texture. At the optimal scan speed, an increase in grain size attributed to epitaxial growth was observed, resulting in the formation of a <100> cubic texture. Consequently, a reduction in high-angle grain boundaries (HAGB) was achieved, suppressing defects such as cracks and enhancing relative density up to 98.1%. Furthermore, with increasing densification, Vickers hardness also exhibited a corresponding increase. These findings underscore the efficacy of PBF-LB/M for processing metals with high DBTT properties.

## 1. Introduction

Refractory elements garner attention due to their exceptional thermal stability, corrosion resistance, and high wear resistance at elevated temperatures [1,2,3,4,5,6,7]. Specifically, chromium (Cr) metal has garnered considerable interest within alloy systems owing to its elevated melting point, reduced density, and enhanced resistance to oxidation when compared to nickel-based superalloys [8,9,10]. Furthermore, its elevated hardness, diminished coefficient of friction, and notable corrosion resistance attributable to the formation of Cr oxide (Cr_2_O_3_) film render it applicable for hard chrome plating (HCP) across diverse sectors, including aerospace, oil and gas, automotive, and papermaking industries [11,12,13,14,15,16]. Nevertheless, Cr exhibits a DBTT of about 150 °C [17,18], significantly above ambient temperature, coupled with limited plastic formability even at temperatures surpassing the DBTT threshold, like other refractory elements [19,20]. This limitation not only constrains its processability, but also presents a notable impediment to its utilization in engineering applications. Consequently, the material has been precluded from widespread application in structural material and related domains. Additionally, using toxic and carcinogenic hexavalent Cr (Cr^6+^) to fabricate Cr alloys and coatings results in significant environmental contamination [14,21]. Consequently, the production of Cr metal and Cr alloys utilizing current methodologies is subject to limitations.

Recently, to overcome the DBTT problem of refractory metals, powder bed fusion-laser beam for metals (PBF-LB/M) has garnered considerable attention as a manufacturing technique that surpasses existing methodologies. PBF-LB/M offers the advantage of fabricating intricate components in near-net shapes [22,23,24,25]. Ramakrishnan et al. recently investigated the impact of Ar and N_2_ shielding gases on crack formation in pure W during the PBF-LB/M process. Rapid cooling rates inherent to PBF-LB/M led to increased N_2_ dissolution and hindered O diffusion, consequently mitigating oxide formation and suppressing crack occurrence [26]. Chen et al. examined the effect of incorporating 5 wt.% TaC powder into pure W to mitigate cracking. The addition of TaC powder resulted in grain refinement, suppressed oxide formation, and ultimately mitigated crack occurrence [27]. Ramakrishnan et al. explored the influence of Ar-N_2_ mixture shielding gases on crack formation in molybdenum during the PBF-LB/M process. In environments containing 5% Ar-95% N_2_ and 100% N_2_, the introduction of N into the Mo lattice reduced O diffusion, thereby suppressing oxide formation and mitigating crack occurrence [28]. However, there has been almost no research on pure Cr produced with PBF-LB/M.

For this reason, we focused on PBF-LB/M to produce pure Cr. In addition to the high design freedom of PBF-LB/M, it enables the attainment of distinctive microstructures characterized by high cooling rates (10^5^–10^7^ K/s) and steep temperature gradients, facilitating control over crystallographic textures [29,30,31,32]. Specifically, texture control, encompassing crystal orientation manipulation, emerges as a pivotal determinant influencing material property, alongside mechanical attributes including strength [33,34], ductility [33,34], Young’s modulus [31], and other functional characteristics such as corrosion resistance [35] and high-temperature oxidation behavior [36]. Consequently, the manipulation of crystallographic texture emerges as a pivotal factor directly influencing material properties, prompting an expansion of research endeavors focused on crystallographic texture control via PBF-LB/M. Nevertheless, there is a dearth of research addressing the mechanism underlying crystallographic texture development concerning the process parameters of PBF-LB/M in pure Cr.

In light of the existing research gaps pertaining to the PBF-LB/M of pure Cr, our research group directed its attention towards addressing this deficiency by conducting an investigation into the PBF-LB/M process applied to pure Cr [36,37]. In ref. [36], we analyzed the effect of high-temperature oxidation resistance on crystallographic texture. A 10 mm × 10 mm × 5 mm test piece was produced, and fixed layer thickness (h = 20 µm), laser power (*p* = 250 W), and hatch spacing (d = 80 µm) were used. Scan speed was applied in the range of 500 to 1000 mm/s as a variable, and the plate was pre-heated to 80 °C. The laser scan direction was XY-scan, which was rotated 90° in the next layer. A strong <100> texture was formed, reaching multiples of unity distribution (MUD) of 5.9 due to an increase in energy density owing to a decrease in scan speed, which resulted in an increase in high-temperature oxidation resistance. Next, in ref. [37], the effect of scan length on densification was studied, and test specimens of 10 mm × 10 mm × 5 mm (long scan length) and 5 mm × 5 mm × 5 mm (short scan length) were compared. The same process parameters of layer thickness (h = 20 µm), laser power (*p* = 300 W), hatch spacing (d = 80 µm), and scan speed (v = 600 mm/s) were applied to both specimens. Additionally, the plate was pre-heated to 80 °C, and the laser scan direction was XY-scan, which was rotated by 90° in the next layer. The decrease in scan length generated uniform heat distribution in the short time interval between scan tracks, which reduced residual stress and increased densification while further strengthening the crystallographic orientation.

According to the understanding presented in ref. [36] indicating texture strengthening via an increase in energy density, and based on ref. [37], which exhibited stronger <100> grain alignment with the application of a short scan length to increase densification, this study was designed to investigate the implementation of both strategies (high energy density adjusted by scan speed and short scan length by 5 mm × 5 mm × 5 mm sample size). The investigation delved into the interrelation between crystallographic texture, energy density, microstructure, and defects. In essence, this study proposed avenues for enhancing density, mitigating defects, and refining the crystallographic texture of pure Cr through PBF-LB/M, thus, proposing an approach to overcome the cracking phenomenon and enhance densification for refractory metals and high-entropy alloys.

## 2. Materials and Methods

**Figure 1:**
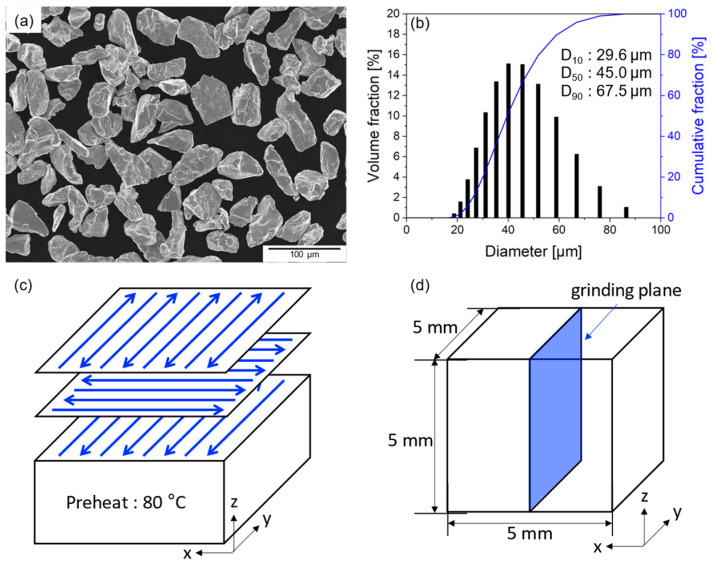
(**a**) Morphology and (**b**) particle size distribution of pure Cr powders, (**c**) schematic representation of the laser scan strategy; blue arrows mean laser scan path, and (**d**) schematic representation of the sample preparation for the observation.

Pure Cr powder (>99% purity) was supplied by JFE Material (Kawasaki, Japan) in an irregular shape, as shown in Figure 1a, which made production more challenging. The particle size distribution of each powder was assessed utilizing a Mastersizer 3000E instrument (Malvern Panalytical, Malvern, UK). The volume weighted percentiles of the Cr powder were D_10_ = 29.6 µm, D_50_ = 45.0 µm, and D_90_ = 67.5 µm (Figure 1b). In this study, pure Cr specimens with dimensions measuring 5 mm (depth) × 5 mm (length) × 5 mm (height) were fabricated utilizing a PBF-LB/M machine (EOS M290, EOS, Krailling, Germany) equipped with a 400 W Yb-fiber laser as the primary heat source, featuring a laser beam size of 100 µm. The manufacturing process adopted an XY-scan strategy with preheating to 80 °C, wherein the scanning direction was rotated 90° between successive layers, as shown in Figure 1c. The scan speed (v) was applied at 400, 600, 800, and 1000 mm/s. Consistent laser power (W) of 300 W, a hatch space (d) of 0.08 mm, and a layer thickness (t) of 0.02 mm were maintained across all conditions (Table 1). The volumetric energy density (*VED*) for the fabrication conditions is defined by Equation (1).

(1) $$VED=\frac{p}{vtd}\;[J/{mm}^{3}]$$

The *VED* calculated using Equation (1) was 468.8 J/mm^3^ for the V400, 312.5 J/mm^3^ for the V600, 234.4 J/mm^3^ for the V800, and 187.5 J/mm^3^ for the V1000.

We cut the as-built test specimen using a wire electrical discharge machine (WEDM, Brother, Nagoya, Japan, HS-300) to observe its y-z plane. The cross-section of the center of the specimen was ground using SiC papers #400–2000 (SANKYO RIKAGAKU Co.,Ltd., Saitama, Japan) and then polished using 0.06 µm colloidal silica.

The relative densities (relative density% = 100% − crack density%) of specimens produced via PBF-LB/M were assessed using an optical microscope (OM; BX-60, Olympus, Tokyo, Japan) in the y-z plane. The relative density of each sample was measured using ImageJ software (version 1.53 k). The specimen underwent etching with a mix of 15 mL of HNO_3_ and 15 gr ceric ammonium nitrate in 80 mL of water to facilitate observation of the morphology of the melt pool in the y-z plane as well as the cellular structure within the melt pool. Microstructural features and crystallographic textures were investigated utilizing a field-emission scanning electron microscope (FE-SEM; JIB-4610F, JEOL, Akishima, Japan) equipped with an electron backscatter diffraction system (EBSD; NordlysMax^3^, Oxford Instruments, Abingdon, UK) in the y-z plane. The microhardness of pure Cr specimens in their as-built state was assessed along the y-z plane employing a micro Vickers tester (HMV-G, Shimadzu, Kyoto, Japan) with a 4.903 N load applied for 20 s. The resultant average value was derived from 10 measurements.

## 3. Results

**Figure 2:**
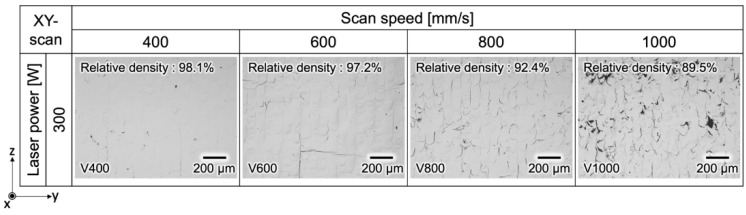
OM images of the y-z cross-section of the as-fabricated PBF-LB/M specimens.

Figure 2 shows OM images depicting the y-z planes of specimens V400, V600, V800, and V1000. The V1000 sample exhibited the lowest relative density at 89.5%. Conversely, for V800 and V600, for which the scan speed was reduced, the *VED* increased, resulting in a rise in relative density to 92.4% and 94.9%, respectively. V400 demonstrated the highest relative density at 98.1%, representing the pinnacle of relative density achieved in pure Cr fabricated via PBF-LB/M when compared with previous reports [36,37]. Defects, such as considerable cracks and lack of fusion, were observed in samples of V1000. This observation suggests that the *VED* employed was inadequate, and thus unable to form a stable melt pool for fabricating pure Cr with its high melting point. Therefore, increasing *VED*, or else decreasing scan speed, promoted densification by eliminating the lack of fusion defects owing to increased remelting between the subsequent layers. Moreover, implementing the lowest scan speed for V400 significantly suppressed cracks and lack of fusion defects while ensuring sufficient *VED* for uniform and sufficient melting, with a wider and deeper melt pool. Furthermore, the reduction in cracking observed in V400 can be attributed to the decrease in high-angle grain boundaries (HAGB) owing to epitaxial growth, a topic to be elaborated upon in subsequent sections.

**Figure 3:**
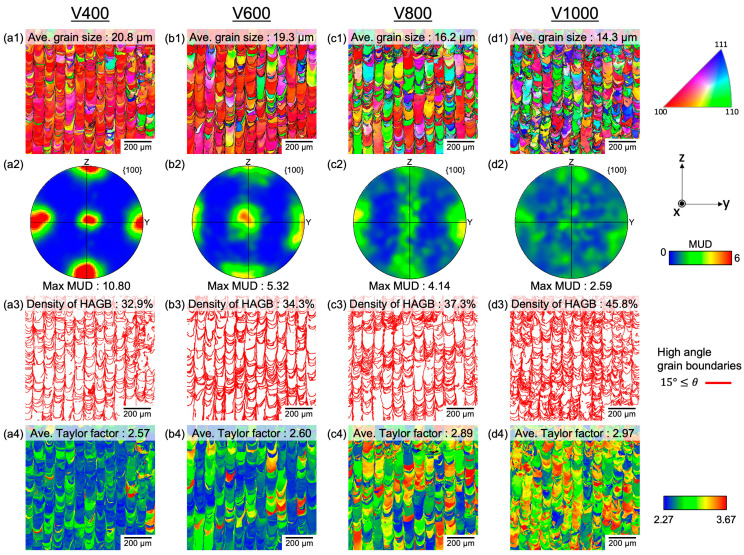
(**a1**–**d1**) IPF maps along BD and (**a2**–**d2**) {100} pole figures of the y-z plane orientation in the Z-direction. (**a3**–**d3**) Corresponding high-angle grain boundary maps and (**a4**–**d4**) Taylor factor maps.

Figure 3 presents EBSD results delineating the impact of scan speed on microstructure. Figure 3 includes an inverse pole figure (IPF) map along the z direction (Figure 3(a1–d1)) and the corresponding {100} pole figure (Figure 3(a2–d2)), HAGB map (Figure 3(a3–d3)), and Taylor factor maps (Figure 3(a4–d4)). In the IPF map, the <100> orientation is represented by red, the <110> orientation by green, and the <111> orientation by blue. Various color patterns were observed in the IPF map of V1000, indicating a random crystalline texture. However, as the scanning speed decreased and the *VED* increased, according to Equation (1), the red area of the IPF map expanded, and most parts of the IPF maps for V400 showed red. The expansion of the red area signifies that as the *VED* rose due to decreased scanning speed, the crystallographic texture aligned towards the {100} orientation. PF further corroborates these findings. The <100> PF of V1000 demonstrated the lowest MUD value (MUD_V1000_: 2.59). Conversely, as the scanning speed decreased, the MUD value escalated, with V400 exhibiting the highest MUD (MUD_V400_: 10.80, MUD_V600_: 5.32, MUD_V800_: 4.14), while previous reports were able to reach UD: 6.2 at best. In this study, V1000 exhibited a random crystallographic texture, V800 demonstrated a fiber texture rotating between the x and z axes with <100> aligned along the *y*-axis, V600 manifested a weak <100> cubic texture with <100> orientation aligned along the x, y, and z axes, and V400 displayed a strong <100> cubic texture with <100> orientation aligned along the x, y, and z axes. Additionally, it is noteworthy that the average grain size increased (V1000: 14.3 µm, V800: 16.2 µm, V600: 19.3 µm, V400: 20.8 µm) with ascending crystallographic texture, as shown in Figure 3(a1–d1) and Figure 3(a2–d2). This phenomenon can be attributed to epitaxial growth across the melt pool, which led to less misorientation between neighboring grains. Accordingly, the density of HAGB, which is susceptible to crack initiation and propagation, also decreased, showing the lowest HAGB density in V400, as shown in Figure 3(a3–d3)) (V1000: 45.8%, V800: 37.3%, V600: 34.3%, V400: 32.9%). The Taylor factor depicted in Figure 3(a4–c4) is a geometric indicator of the efficiency of crystallographic shear in accommodating macroscopic deformations [38,39]. The Taylor factor of high-speed fabrications exhibited high value with the grains observing high residual stress (red colored), suggesting the existence of a high density of dislocation and less capability for further deformation. This created potential crack initiation and propagation sites in the component. Remarkably, a decrease in scan speed (increase in *VED*) corresponded with a reduction in the Taylor factor (V1000: 2.97, V800: 2.89, V600: 2.60, V400: 2.57), indicative of less remaining stress in the grains and enhanced capacity to accommodate deformation.

**Figure 4:**
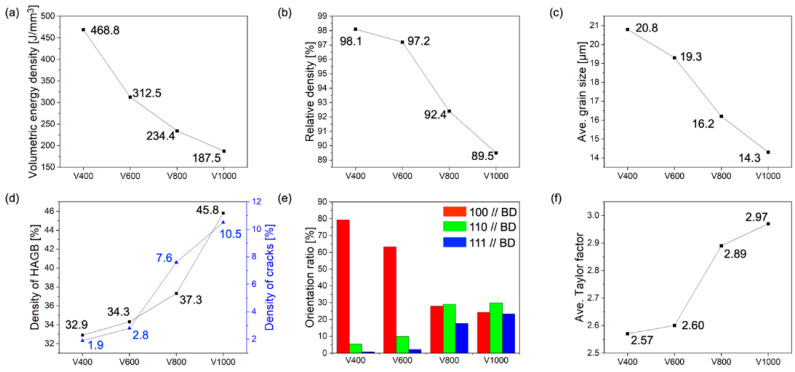
Plots of (**a**) volumetric energy density, (**b**) relative density, (**c**) average grain size, (**d**) density of HAGB and crack density, (**e**) orientation ratio, and (**f**) average Taylor factor from V400, V600, V800, and V1000 samples.

Figure 4 shows various parameters, including (a) *VED*, (b) relative density, and (c) average grain size obtained from EBSD analysis, as well as (d) density of HAGB and crack density, (e) orientation ratio, and (f) average Taylor factor, elucidating their correlations. Notably, *VED* exhibited an increase as scan speed decreased by Equation (1), resulting in reduced cracks and lack of fusion, which led to an augmentation in relative density. This increase in relative density can be attributed to the decrease in HAGB density facilitated by the enlargement of average grain size. This phenomenon suggests the occurrence of epitaxial growth with escalating *VED*, as corroborated by the orientation ratio depicted in Figure 4e. Specifically, in the V1000 test specimen, the <100>, <110>, and <111> orientations were distributed in similar proportions, indicative of a random crystallographic texture (fraction of <100> orientation in V1000: 24.2%). Conversely, in the V400 test specimen, a predominant orientation along the <100> orientation was observed (fraction of <100> orientation in V400: 97.4%) (Table 2), resulting in a reduction in HAGB density and, consequently, a decreased crack density (V1000: 10.5%, V800: 7.6%, V600: 2.8%, V400: 1.9%). This observation aligns with previous studies, indicating that HAGB is the predominant source of cracks in PBF-LB/M [36,40,41]. Further elucidation of the detailed crystallographic texture formation mechanism is deferred to subsequent sections. The Taylor factor indicates grains’ susceptibility to deformation during plastic deformation; a higher Taylor factor denotes increased resistance to plastic deformation [42]. Remarkably, the V400 test specimen exhibited the lowest average Taylor factor, which was attributed to diminished initial plastic deformation owing to higher laser energy decreasing the residual stress. Thus, a reduction in scan speed facilitated an increase in *VED*, thereby decreasing stress while promoting epitaxial growth between build layers, and ultimately leading to a decrease in HAGB density, a reduction in cracks, and an increase in relative density.

**Figure 5:**
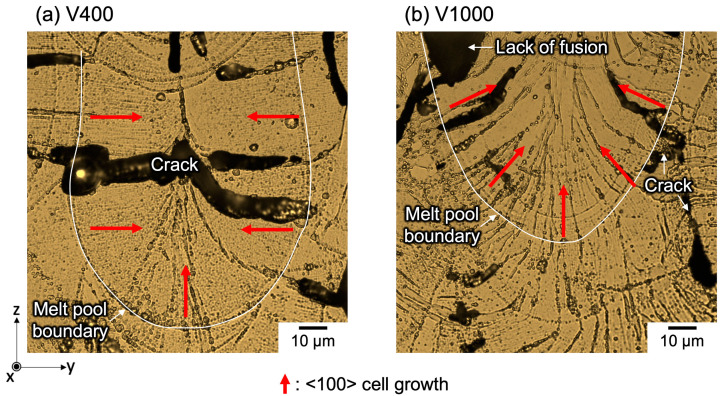
Etched melt pool cross-section in the y-z plane showing the cellular-type microstructure grown by OM.

Figure 5 illustrates the variation in melt pool morphology concerning *VED* alterations in the y-z plane of pure Cr processed via PBF-LB/M. A comparative analysis was conducted between the V400 sample, characterized by the most pronounced <100> cubic texture, and the V1000 sample, which exhibited a random texture. Etching with a mix of nitric acid and ceric ammonium nitrate was employed to inspect the cellular microstructure within the melt pool. In the case of V400, a narrow and deep melt pool formation was evident, and attributed to the heightened *VED* resulting from reduced scan speed (Figure 5a). Conversely, V1000 exhibited a broader and shallower melt pool due to reduced *VED* resulting from increased scan speed (Figure 5b). Both specimens displayed cracks attributable to the high DBTT of pure Cr. However, a lack of fusion was exclusively observed in V1000, but not in V400. This disparity can be attributed to the increased *VED* accompanying decreased scan speed, which led to deeper melt pool formation and enhanced layer overlap in V400. Conversely, in V1000, decreased *VED* associated with increased scan speed resulted in a shallow melt pool, precluding adequate layer overlap. Furthermore, the lower *VED* in V1000 proved inadequate for achieving complete melting of high melting point pure chromium. In terms of microstructural features, a cellular-type microstructure manifested perpendicular to the *z*-axis (building direction (BD)), while a directional growth was observed along the BD at the bottom of the melt pool boundary in V400. Conversely, in V1000, a cellular-type microstructure developed perpendicular to the melt pool boundary. The disparity in growth directions of this cellular-type microstructure contributed to differences in crystallographic texture formation, a subject of subsequent discussion.

## 4. Discussion

### 4.1. Texture Formation Mechanism

**Figure 6:**
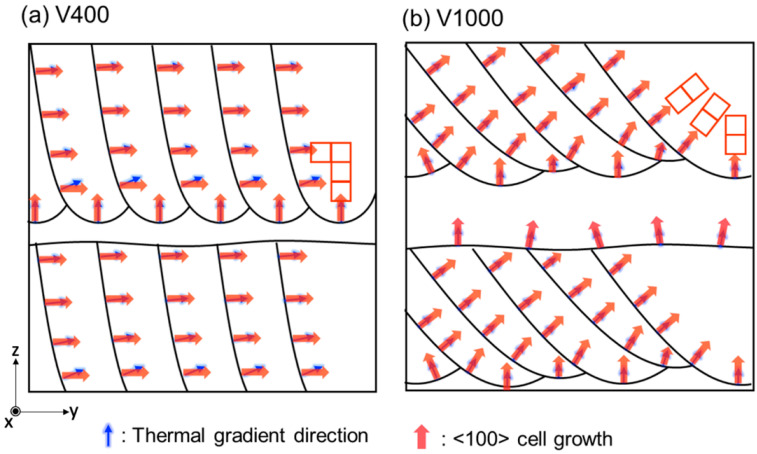
Schematics of the mechanism for texture evolution depending on scan speed.

Figure 6 illustrates a schematic diagram of the melt pool shapes in the y-z plane elucidating the texture formation mechanism of pure Cr via PBF-LB/M corresponding to the difference in laser scan speed as well as *VED*. Depending on the scan speed, a transition from a random texture to a <100> cubic texture was observed for additively manufactured pure Cr. This phenomenon arose from the interplay between the melt pool shape and the direction of the thermal gradient (blue arrow). Specifically, the V1000 specimen, characterized by a rapid scan speed and low *VED*, was anticipated to form a wide, shallow, and unstable melt pool, therefore resulting in random texture formation. Pure Cr in the BCC structure exhibits a preference for grain growth along the <100> direction (being the less atomically dense lattice plane, which requires less energy to grow) [43]. However, when oriented in alignment with the thermal gradient direction perpendicular to the melt pool [44] under conditions of low *VED*, growth into columnar grains becomes complicated due to the reduced thermal gradient with varied direction compared to high *VED* conditions [29]. Furthermore, the application of the XY-scan strategy, involving a 90° rotation in scanning direction for the subsequent layer, led to a mismatch in the grain growth direction between layers, precluding epitaxial growth across the melt pool and resulting in a random crystallographic texture (Figure 3(d2) and Figure 4e).

Conversely, in the case of the V400 specimen with a slower scan speed, a deep and stable melt pool formed owing to increased *VED*. Additionally, the heightened *VED* resulted in an increased thermal gradient, facilitating growth into columnar grains. Along the center of the melt pool, the thermal gradient aligned with the *z*-axis direction (BD), encouraging grains oriented <100> to grow along this direction. Despite discrepancies between the thermal gradient direction and the <100> axis of the substrate caused by curvature along the side of the melt pool, growth in the <100> direction prevailed, as it required less energy than initiating new grain growth [32,45]. In subsequent layers, where the scanning direction rotated by 90°, grains continued to grow in the <100> direction, enabling epitaxial growth across the melt pool and resulting in a <100> cubic texture (Figure 3(a2) and Figure 4e). Consequently, V400 exhibited bigger grain size, less HAGB, and microstructure more prone to crack during initiation and propagation, owing to reduced residual stress.

### 4.2. Hardness Response to Defects and Texture

**Figure 7:**
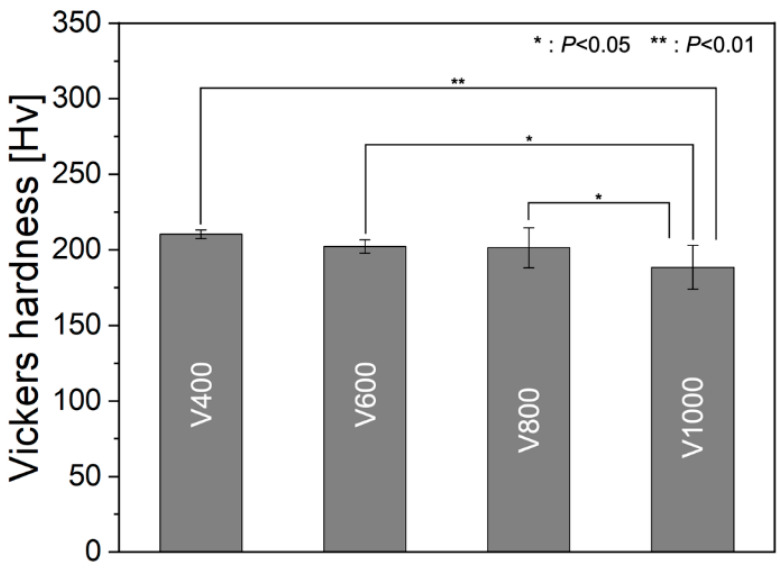
Vickers hardness of pure Cr specimens fabricated via PBF-LB/M in the average of 10 measurements. *: *p*  <  0.05, **: *p*  <  0.01 by Tukey’s test.

Figure 7 depicts the influence of scan speed on Vickers hardness. As scan speed decreased and energy density increased, hardness exhibited a corresponding increase, with the V400 test specimen demonstrating the highest hardness value and the slightest error bar (Table 3). This observed trend aligns with the trend observed in relative density, thus indicating a decrease in defects [46,47,48]. Notably, the V1000 specimen, characterized by the lowest relative density, exhibited the lowest hardness and displayed the most extensive error bar, attributable to limited reproducibility and the existence of excessive defects, such as cracks and lack of fusion. Conventionally, smaller grain sizes, represented by high scan speed (V1000) in this study, correlate with higher strength and hardness, as per the Hall–Petch Equation. Because the grain boundary exhibits significantly greater disorder compared to the interior of the grain, it thereby impedes the uninterrupted movement of dislocations along a continuous slip plane. Alongside grain size, the Taylor factor plays a pivotal role in determining mechanical strength, given its association with the resistance to deformation induced by external loads [39]. In the context of polycrystalline metallic materials, the phenomenon of dislocation movement is referred to as slip, which holds significance in understanding deformation behavior. The assessment of slip in polycrystalline materials is facilitated through the Taylor factor, which entails the averaging of grain orientations across the entirety of grains within the specimen [49]. This parameter serves to forecast the requisite work for deformation or distortion. High Taylor factor values associated with a grain indicate the heightened stress threshold required to initiate slip, consequently augmenting the material’s strength or hardness [42]. Moreover, within a textured polycrystalline material, the Taylor factor offers insight into the comparative strength of an individual crystal based on its orientation [50]. The influence of grain orientation on the Taylor factor manifests distinctly, as it is derived from cumulative slips across various slip systems within the grain [51]. In this study, as scan speed increased, the average Taylor factor increased (Figure 3(a4–d4) and Figure 4f). The Taylor factor increased in V1000, which showed a random crystallographic texture. The elevated Taylor factor noted in V1000 samples can be attributed to the irregular random texture observed therein, fostering a more significant involvement of crystallographic orientations in competitive grain growth dynamics (Figure 3(d1–d4)). However, contrary to this expectation, the V1000 sample, possessing the smallest grain size due to lack of epitaxial growth (which resulted in random texture), exhibited the lowest hardness, suggesting a heightened susceptibility to defects. Conversely, a significant increase in hardness was noted in the V400 specimen, wherein a reduction in HAGB and reduced defects was achieved through epitaxial growth, underscoring the efficacy of crystallographic texture development, thus presenting promising results for the producibility and applicability of textured pure Cr via the PBF-LB/M process. However, the hardness value in this study was lower than the previously reported hardness value of HCP (734–1097 Hv) [11,15]. In the case of pure Cr produced via PBF-LB/M in this study, approximately 5 at.% C and 3 at.% O were observed under all conditions. Our study contained lower interstitials than reports that achieved solid solution strengthening using interstitials such as C [52]. Among other reasons, this was because HCP has a small grain size of about 50 nm or less [53]; it was also due to the Hall–Petch effect due to the grain size smaller than pure Cr (14.3–20.8 µm) produced via PBF-LB/M. In order to strengthen the competitiveness of pure Cr produced via PBF-LB/M, higher density and hardness are required.

## 5. Conclusions

This study investigated the influence of scan speed on the density and crystallographic texture of pure Cr produced via PBF-LB/M. In the V400 specimen, fabricated with a slower scan speed and an XY-scan strategy via PBF-LB/M, an enlargement in average grain size and a reduction in the proportion of crack-prone HAGB were observed and attributed to the prominent <100> cubic texture and simultaneously reduced residual stress. Consequently, a heightened relative density was achieved. These findings underscore the potential of crystallographic texture control as a strategy to mitigate defects in metals and alloys with high DBTT, such as Cr. Moreover, achieving a higher relative density to satisfy commercial needs is imperative for industrial applications, necessitating further research in this domain.

## Figures and Tables

**Table 1:** Process parameters, including laser power, scan speed, hatch space, layer thickness, and *VED* of pure Cr specimens fabricated via PBF-LB/M.

	Laser Power [W]	Scan Speed [mm/s]	Hatch Space [mm]	Layer Thickness [mm]	*VED* [J/mm^3^]
V400	300	400	0.08	0.02	468.8
V600		600			312.5
V800		800			234.4
V1000		1000			187.5

**Table 2:** Orientation ratio of <100>, <110>, and <111> along building direction (BD) from V400, V600, V800, and V1000 samples.

	Fraction of <100> [%]	Fraction of <110> [%]	Fraction of <111> [%]
V400	79.4	5.35	0.82
V600	63.2	9.91	2.24
V800	27.9	29.1	17.6
V1000	24.2	29.9	23.3

**Table 3:** Vickers hardness values from as-built pure Cr specimens.

	Hardness [Hv]
V400	210.3 ± 2.9
V600	202.2 ± 4.4
V800	201.4 ± 13.3
V1000	188.5 ± 14.4

## Data Availability

Data supporting the findings of this study are available from the corresponding author upon reasonable request.
